# Complete mitochondrial genome and phylogenetic analysis of *Schizothorax molesworthi* (Cypriniformes: Cyprinidae)

**DOI:** 10.1080/23802359.2017.1407714

**Published:** 2017-11-27

**Authors:** Chi Zhang, Chaowei Zhou, Tingbing Zhu, Benhe Zeng, Baohai Li

**Affiliations:** a Institute of Fisheries Science, Tibet Academy of Agricultural and Animal Husbandry Sciences, Lhasa, PR China;; b Department of Aquaculture, Southwest University Rongchang Campus, Chongqing, PR China;; c Yangtze River Fisheries Research Institute, Chinese Academy of Fisheries Science, Wuhan, China

**Keywords:** *Schizothorax molesworthi*, mitochondrial genome, phylogenetic

## Abstract

*Schizothorax molesworthi* is an endemic species and distributes in the MoTuo reaches of the Yarlung Zangbo River. It is one of the most important commercial fishes in this area. In the present study, the complete mitochondrial DNA sequence of *Schizothorax molesworthi* was determined and analyzed. The mitochondrial genome of *Schizothorax molesworthi* is 16,585 bp in length and consisted of 37genes in the typical vertebrate mitochondrial gene arrangement. Overall base composition of mitochondrial genome of *Schizothorax molesworthi* was 30.1% A, 26.9% C, 17. 4% G, and 25.6% T, with a high A + T content (55.7%). Phylogenetic analysis showed that *Schizothorax molesworthi* and *Schizothorax plagiostomus* clustered together in a clade and formed a sister relationship.

*Schizothorax molesworthi* is categorized into subfamily Schizothoracinae, family Cyprinidae, order Cypriniformes. It is restrict distributed in the MoTuo reach of the Yarlung Zangbo River, southwest China (Wu and Wu 1992). It is a valued economic fish in this region and characterized by low growth rate and late sexual maturity. The natural population of *Schizothorax molesworthi* has been dramatically decreased during recent years. Currently, *Schizothorax molesworthi* is defined as a vulnerable species by Vertebrate red list in China (Jiang et al. 2016). Here, we report the complete mitogenome sequence of *Schizothorax molesworthi*, which can be used in the studies on molecular systematics, phylogeography, stock evaluation, and conservation genetics.

The sample of *Schizothorax molesworthi* was obtained from MoTuo (29°15′–29°21′N; 95°11′–95°22′E) at an altitude of 900 m in Tibet, China. This place has an exceptional aquatic ecosystem, unique geology, topography and climate. The whole body specimen was preserved in ethanol and registered to the Specimen Depository, Fisheries Research Institute, Tibet Academy of Agricultural and Animal Husbandry Sciences. The total genomic DNA was extracted from the pelvic fin preserved in 95% alcohol. According to the mtDNA sequence of Schizothorax waltoni (GenBank accession no. KC513574), 20 primers were designed to amplify the PCR products for sequencing. Mitochondrial genome was annotated using MitoAnnotator (Iwasaki et al. 2013). Gene map of the complete mitochondrial genome was performed using Dual Organellar GenoMe Annotator (DOGMA) (Lohse et al. 2013). MEGA 7.0 software was used for calculating the base composition and constructing a maximum-likelihood (ML) tree (Kumar et al. 2016).

The mitogenome of *Schizothorax molesworthi* was a circular molecule of 16,585bp in length (GenBank accession no. MG171194)It consists of 13 protein-coding genes (PCGs), 2 rRNAs, 22 tRNAs and 2 non-coding regions: origin of light-strand replication (O_L_) and control region. Except for eight tRNAs (Gln, Ala, Asn, Cys, Tyr, Ser, Glu, and Pro) genes and one protein-coding gene (ND6), most of the genes were encoded on the heavy strand (H-strand).which shows a typical gene arrangement of vertebrate mitogenomes (Anderson et al. 1981).

The overall base composition of the mitogenome is 30.1% for A, 26.9% for C, 17.4% for G and 25.6% for T, with a high A + T content (55.70%), indicating an obvious anti-guanine bias commonly observed in teleost fishes (Jondeung et al. 2007). The length of 22 tRNA genes varies from 67 bp (tRNA Cys) to 76 bp (tRNA Leu and tRNA Lys). The 12S rRNA and 16S rRNA are 956 bp and 1675 bp, respectively. These two rRNA genes are located in the typical positions between tRNA Phe and tRNA Leu and separated by tRNA Val. A major non-coding region between the tRNA Pro and tRNA Phe genes (936 bp) is considered to be the control region (D-loop).

The phylogenetic tree (Figure 1) was constructed by the maximum-likelihood methods using complete mitochondrial genomes of 15 Schizothoracinae species. The tree supports clear phylogenetic relationships at the genus level. The results of the present study could serve as a basis for future studies on the taxonomic resolution and phylogenetic relationships of Schizothoracinae.

**Figure 1.**
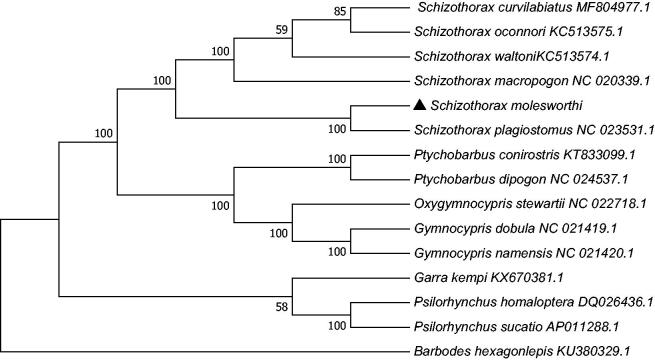
Phylogenetic tree based on the complete mitochondrial genome sequences was constructed by maximum-likelihood (ML) analysis with Kimura 2-parameter method with 500 bootstrap replicates. GenBank accession numbers of mitogenomic sequences for each taxon are shown in parentheses.
